# Hypertriglyceridemic-waist phenotype predicts diabetes: a cohort study in Chinese urban adults

**DOI:** 10.1186/1471-2458-12-1081

**Published:** 2012-12-15

**Authors:** Meilin Zhang, Yuxia Gao, Hong Chang, Xuan Wang, Dongmei Liu, Zongjian Zhu, Guowei Huang

**Affiliations:** 1 Department of Nutrition and Food Hygiene, School of Public Health, Tianjin Medical University, Tianjin, China; 2 Department of Cardiology, General Hospital of Tianjin Medical University, Tianjin, China; 3 Health Examination Center of Heping District, Tianjin, China

## Abstract

**Background:**

Hypertriglycedemic-waist (HTGW) phenotype is a simple and inexpensive screening parameter to identify people at increased risk for cardiovascular disease. We evaluated whether the HTGW phenotype predicts prediabetes and diabetes in Chinese urban adults.

**Methods:**

Two thousand nine hundred and eight (2908) subjects including 1957 men and 951 women, aged 20 years and older, free of prediabetes and diabetes at baseline were enrolled in 2008 and followed for 3 years. Meanwhile, new cases of prediabetes and diabetes were identified via annual physical examination. Cox proportional hazards models were used to assess the association of HTGW phenotype with the incidence of prediabetes and diabetes.

**Results:**

One thousand five hundred and thirty-three (1533) new prediabetes and 90 new diabetes cases were diagnosed during the follow-up period. The accumulated incidence of prediabetes and diabetes was 52.7% and 3.1%, respectively. Compared with the normal waist normal triglyceride (NWNT) group, those in the HTGW group had higher incidence of prediabetes and diabetes for both men and women. The hazard ratio (HR) for developing prediabetes in the presence of HTGW phenotype at baseline was 1.51 (95% confidence interval [CI] =1.04-2.19) in women, not in men (HR=1.01; 95% CI = 0.82-1.24), after adjusting for age, body mass index, systolic blood pressure, total cholesterol and low density lipoprotein-cholesterol. The HR for developing diabetes were 4.46 (95% CI = 1.88-10.60) in men and 4.64 (95% CI = 1.20-17.97) in women for people who were HTGW phenotype at baseline, after adjusting for age, body mass index, systolic blood pressure, total cholesterol and low density lipoprotein-cholesterol.

**Conclusions:**

The HTGW phenotype can be used as a simple screening approach to predict diabetes. By using this approach, it is possible to identify individuals at high-risk for diabetes, which is of great significance in reducing the incidence of diabetes among Chinese urban adults.

## Background

Cardiovascular disease (CVD) has been a leading cause of death in China [1]. Diabetes and prediabetes are the major risk factors for the cardiovascular disease, and the prevalence of diabetes and prediabetes in Chinese adults has been elevated to 9.7% and 15.5%, respectively [2]. Although the prevalence of diabetes and prediabetes in China remains lower than that in the industrialized countries, the enormous population in China, estimated to be over 1.3 billion, makes the largest population with diabetes in the world. A national survey conducted in 2007–2008, involving 46,239 Chinese adults showed that there were 92.4 million Chinese suffering from diabetes (50.2 million men and 42.2 million women), with an additional 148.2 million living with prediabetes (76.1 million men and 72.1 million women) [2].

The American Diabetes Association (ADA) refers to patients with impaired fasting glucose (IFG) as having “pre-diabetes”, which indicates the relatively high risk for development of diabetes in these patients [3]. With the high prevalence of IFG, the prevalence of diabetes could continue to increase rapidly in China. Recently, International Diabetes Federation (IDF) has estimated the number of people worldwide with diabetes for 2011 and 2030 and shows that the global diabetes epidemic continues to grow [4]. By 2030, the number of individuals with diabetes worldwide is expected up to 552 million and most people with diabetes live in low-and middle-income countries where the diabetes drugs and insulin are often inaccessible or too expensive, and the local health-care systems do not have the capacity deal with personnel and financial issues.

As diabetes is becoming a serious threat to human being’s health, using a simple and inexpensive screening method for early diagnosis is particularly important [5,6]. The Third Report of the National Cholesterol Education Program Expert Panel (NCEP ATP III) suggested that abdominal obesity is an independent risk factor for diabetes and measuring waist circumference (WC) is an effective tool to screen individuals at high-risk of diabetes [7,8]. However, because WC cannot fully discriminate intra-abdominal from subcutaneous abdominal adiposity, the elevated triglyceride levels have been adopted as a marker of “dysfunctional” adipose tissue, intra-abdominal obesity and associated metabolic abnormalities in people with an increased waistline [9-11]. Therefore, the concept of hypertriglycedemic-waist (HTGW) phenotype has been used as a simple and inexpensive screening approach to identify people at increased risk of cardiovascular disease [12-14]. Lemieux et al. [10] were the first group to recognize that the HTGW phenotype was associated with increased CVD risk in men. In particular, the HTGW was associated with the atherogenic triad of hyperinsulinemia, elevated concentrations of apolipoprotein B and small, dense low density lipoprotein-cholesterol (sdLDL-C) particles. In corroboration, HTGW is associated with increased CVD risk in women [13]. After 7.5 years of follow-up in a low-risk middle-aged men population, HTGW was associated with the risk of CVD [15].

Although abdominal obesity and hypertriglyceridemia have been known as risk factors for diabetes, limited data are available in the linkage of HTGW phenotype to prediabetes and diabetes. Currently, only one study has shown that the phenotype of abdominal obesity HTGW was highly prevalent in Chinese adults, 35.4% in the women, 33.6% in the men, and the phenotype of visceral obesity HTGW was closely associated with prediabetes and diabetes [16]. However, it is a cross-sectional study in which it is difficult to derive HTGW from etiology of prediabetes and diabetes. Therefore, we conducted a cohort study to evaluate whether HTGW, as the phenotype of visceral obesity, was closely associated with the risk of prediabetes and diabetes among Chinese urban adults.

## Methods

### Study population

In 2008, we recruited 4238 individuals from the Health Examination Center of Heping District in Tianjin, China. Persons aged 20 years and older were eligible and were enrolled when they participated in their annual health examination. At baseline, 848 subjects with prediabetes (5.6 ≤ fasting glucose <7.0 mmol/L) and 482 subjects with diabetes (fasting glucose ≥7.0 mmol/L) or with a history of diabetes were excluded from the enrollment. Totally, the baseline survey recruited 2908 persons (1957 men and 951 women) who did not have prediabetes and diabetes or free of a history of diabetes. Because many participants were expected to have repeated examinations, we took advantages of this opportunity to conduct a follow-up study on prediabetes and diabetes. All participants repeated the health examination in 2011 and their records included complete data from clinical and laboratory measurements.

The study protocol was approved by the ethics committee of Tianjin Medical University and informed consent was signed by each participant.

### Clinical and laboratory measurements

Body weight of the participants who only wore light clothing without shoes was assessed by a trained staff using digital scales and recorded to the nearest 0.1 kg. The height was measured using tape meter when the bare foot participants were standing with shoulders in normal alignment. Body mass index (BMI) was calculated as weight in kilograms divided by the square of the height in meters (kg/m^2^). Waist circumference was measured at the level of the umbilicus, using an unstretched tape meter, without any pressure to body surface over light clothing. Two measurements of systolic blood pressure (SBP) and diastolic blood pressure (DBP) were taken using a standardized mercury sphygmomanometer on the right arm, after a 15-minute rest in a sitting position; the average of the two measurements was used as subject’s blood pressure. A blood specimen was collected after overnight fasting into a vacuum tube containing sodium fluoride for measurement of plasma glucose and lipid profiles. Plasma glucose and lipid levels were determined using automatic clinical analyzer (TBA-40, Japan) [17]. The measurements of the lipid profiles were standardized according to the criteria of the Centers for Disease Control and Prevention-National Heart, Lung, and Blood Institute Lipid Standardization Program [18].

### Definition of terms

Subjects were categorized into 4 phenotype groups based on the following cut-off points: (1) NWNT (normal waist normal triglyceride, waist circumference <90 cm for men and <80 cm for women; serum triglyceride concentration <1.7 mmol/L); (2) HTG (hypertriglyceridemia, waist circumference <90 cm for men and <80 cm for women; serum triglyceride concentration ≥1.7 mmol/L); (3) EW (enlarged waist, waist circumference ≥90 cm for men and ≥80 cm for women; serum triglyceride concentration <1.7 mmol/L); (4) HTGW (hypertriglyceridemic-waist, waist circumference ≥90 cm for men and ≥80 cm for women; serum triglyceride concentration ≥1.7 mmol/L).

The levels of fasting plasma glucose were classified according to the ADA diagnostic criteria as follows [19]: IFG (5.6 ≤ fasting glucose <7.0 mmol/L) as an indicator of prediabetes and diabetes (fasting glucose ≥7.0 mmol/L).

### Statistical analysis

Statistical analyses were performed using SPSS (version 13.0, Chicago, IL, USA). Data were analyzed separately for men and women and the continuous variables of the subjects at baseline were expressed as mean and standard deviation or median and inter-quartile range dependent on the data distribution and analyzed using Analysis of Variance (ANOVA) test with Bonferonni post hoc comparison among the groups of prediabetes (IFG), diabetes (DM) and normal fasting glucose (NFG), or among the 4 phenotype groups. The categorical variables were presented as percentages. Collinearity Diagnostics was used to analyze the multicollinearity among the covariates. The cumulative incidences of prediabetes and diabetes for each phenotype group were analyzed using the Kaplan-Meier method. The Cox proportional hazards models was used to analyze the risk of developing prediabetes and diabetes with and without adjustment for covariates. With the Collinearity Diagnosis, it was observed that SBP, DBP, total cholesterol (TC) and low density lipoprotein-cholesterol (LDL-C) strongly affected each other. Thus, the covariates for adjustment in Cox regression analysis were age, BMI, SBP, TC and high density lipoprotein-cholesterol (HDL-C). The results are expressed as hazards ratios (HRs) and 95% confidence interval (CI). All reported P-values were two-tailed, and P <0.05 was considered statistically significant.

## Results

The baseline characteristics associated with the development of prediabetes and diabetes during the 3 year period are shown in Table 1. There were 2908 individuals in total (1957 males and 951 females) without prediabetes and diabetes at baseline who participated in the follow-up survey. Of these, 1190 men and 343 women developed prediabetes, 64 men and 26 women developed diabetes during the three-year period. The accumulated incidences of prediabetes and diabetes were 52.7% and 3.1%, respectively (data was not shown). The accumulated incidence of prediabetes was significantly higher in men (60.8%) than in women (36.1%) (P< 0.001). The accumulated incidence of diabetes was 3.3% and 2.7% in men and women, respectively, with no statistically significant differences between the genders (P >0.05). Individuals with prediabetes and diabetes tended to be older, have higher body mass index, waist circumferences, fasting glucose levels, triglycerides levels, systolic blood pressures, and diastolic blood pressures in men and women. 

**Table 1:** Baseline characteristics in relation to the development of prediabetes and diabetes

	**Men**				**Women**			
	NFG	IFG	DM	P value*	NFG	IFG	DM	P value*
No. of subjects	703	1190	64		582	343	26	
Age (years)	47.2±15.5	52.1±14.6 ^**#**^	54.8±14.0 ^**#**^	<0.001	42.9±12.8	52.4±12.8 ^**#**^	50.5±14.5 ^**#**^	<0.001
BMI (kg/m^2^)	24.0±3.3	25.2±3.2 ^**#**^	26.4±2.8 ^**#&**^	<0.001	22.2±3.0	23.8±3.6 ^**#**^	25.5±5.0 ^**#&**^	<0.001
WC (cm)	82.1±8.4	85.0±8.4 ^**#**^	90.0±6.0 ^**#&**^	<0.001	73.2±8.2	78.5±8.9 ^**#**^	83.3±11.6 ^**#&**^	<0.001
SBP (mm Hg)	120.8±14.8	126.2±16.4 ^**#**^	133.2±18.3 ^**#&**^	<0.001	111.9±15.6	121.4±17.9 ^**#**^	129.0±19.0 ^**#&**^	<0.001
DBP (mm Hg)	79.3±9.6	81.6±9.7 ^**#**^	84.1±11.5 ^**#**^	<0.001	74.8±9.5	78.8±9.0 ^**#**^	82.5±10.3 ^**#**^	<0.001
TC (mmol/L)	4.6±0.7	4.8±0.8 ^**#**^	4.9±1.1 ^**#**^	<0.001	4.6±0.6	4.8±0.7 ^**#**^	4.7±0.8	<0.001
LDL-C (mmol/L)	2.7±0.6	2.8±0.7 ^**#**^	2.8±0.6	0.002	2.5±0.6	2.7±0.7 ^**#**^	2.5±0.7	<0.001
HDL-C (mmol/L)	1.3±0.3	1.3±0.3	1.2±0.3 ^**&**^	0.038	1.4±0.3	1.4±0.3 ^**#**^	1.3±0.2 ^**#**^	<0.001
TG (mmol/L)	1.2(1.0-1.5)	1.4(1.1-1.8) ^**#**^	1.7(1.4-2.1) ^**#&**^	<0.001	1.0(0.8-1.2)	1.3(1.1-1.6) ^**#**^	1.5(1.2-2.3) ^**#&**^	<0.001
FPG (mmol/L)	4.9±0.4	5.0±0.3 ^**#**^	4.8±0.7 ^**&**^	<0.001	4.8±0.5	5.0±0.4 ^**#**^	4.8±0.5	<0.001

Values are expressed as means ± standard deviation. TG was described by median and inter-quartile range.

Abbreviations: NFG, normal fasting glucose; IFG, impaired fasting glucose; DM, diabetes mellitus; BMI; body mass index; WC, waist circumference; SBP, systolic blood pressure; DBP, diastolic blood pressure; TC, total cholesterol; LDL-C, low density lipoprotein-cholesterol; HDL-C, high density lipoprotein-cholesterol; TG, triglyceride; FPG, fasting plasma glucose.

* Comparison among three groups.

^**#**^ Compared with NFG group, P <0.05.

^**&**^ Compared with IFG group, P <0.05.

Table 2 presents the clinical characteristics and laboratory data stratified by the 4 phenotype groups. Compared with the NWNT group, participants in the groups of HTG, EW and HTGW had a higher body mass index, waist circumference, blood pressure, higher levels of LDL-C and lower levels of HDL-C. This was found for both men and women. For both men and women in the HTGW group, higher levels of fasting plasma glucose were found compared to those in the NWNT group. 

**Table 2:** Baseline clinical characteristics and laboratory data by the 4 phenotype groups

	**NWNT**	**HTG**	**EW**	**HTGW**	**P value**^**a**^
Men					
No. of subjects	1018	428	251	260	
Age (years)	50.1±16.3	50.0±12.9	52.3±15.34	50.6±13.0	NS ^b^
BMI (kg/m^2^)	23.2±2.8	24.6±2.3 ^**#**^	27.8±2.4 ^**#&**^	28.3±2.4 ^**#&**^	<0.001
WC (cm)	79.4±6.4	82.8±4.56 ^**#**^	94.3±4.1 ^**#&**^	95.2±4.6 ^**#&**^	<0.001
SBP (mm Hg)	122.1±15.8	124.6±15.4 ^**#**^	128.5±16.6 ^**#&**^	129.7±16.8 ^**#&**^	<0.001
DBP (mm Hg)	78.8±9.0	81.8±9.7 ^**#**^	83.4±10.3 ^**#**^	85.0±10.3 ^**#&**^	<0.001
TC (mmol/L)	4.6±0.8	5.0±0.8 ^**#**^	4.7±0.7 ^**&**^	4.9±0.8 ^**#$**^	<0.001
LDL-C (mmol/L)	2.6±0.7	2.9±0.7 ^**#**^	2.8±0.6 ^**#**^	2.9±0.6 ^**#**^	<0.001
HDL-C (mmol/L)	1.4±0.3	1.2±0.3 ^**#**^	1.3±0.3 ^**#&**^	1.1±0.2 ^**#&$**^	<0.001
TG (mmol/L)	1.0(0.9-1.2)	2.2(2.0-2.5) ^**#**^	1.2(1.1-1.3) ^**&**^	2.2(2.0-2.4) ^**#$**^	<0.001
FPG (mmol/L)	4.9±0.4	5.0±0.4 ^**#**^	5.0±0.3	5.1±0.3 ^**#**^	<0.001
Women					
No. of subjects	604	67	177	103	
Age (years)	42.5±12.3	49.3±13.7 ^**#**^	52.3±12.8 ^**#**^	58.4±11.4 ^**#&$**^	<0.001
BMI (kg/m^2^)	21.3±2.2	22.4±1.8 ^**#**^	26.2±3.4 ^**#&**^	26.5±3.3 ^**#&**^	<0.001
WC (cm)	70.4±5.1	74.6±3.5 ^**#**^	85.9±6.9 ^**#&**^	86.9±6.7 ^**#&**^	<0.001
SBP (mm Hg)	110.8±15.2	118.9±14.9 ^**#**^	122.9±17.7^**#**^	130.8±16.7 ^**&$**^	<0.001
DBP (mm Hg)	74.1±8.8	79.1±8.8	79.6±9.5 ^**#**^	83.3±9.3 ^**#&$**^	<0.001
TC (mmol/L)	4.5±0.7	4.9±0.7 ^**#**^	4.7±0.7 ^**#**^	5.1±0.7 ^**#$**^	<0.001
LDL-C (mmol/L)	2.4±0.6	2.8±0.6 ^**#**^	2.6±0.6 ^**#**^	2.9±0.7 ^**#$**^	<0.001
HDL-C (mmol/L)	1.5±0.3	1.3±0.3 ^**#**^	1.4±0.3 ^**#&**^	1.2±0.3 ^**#$**^	<0.001
TG (mmol/L)	0.9(0.8-1.1)	2.0(1.9-2.2) ^**#**^	1.2(1.1-1.3) ^**#&**^	2.1(1.9-2.6) ^**#$**^	<0.001
FPG (mmol/L)	4.9±0.5	4.9±0.4	5.0±0.4 ^**#**^	5.0±0.5 ^**#**^	0.006

Values are expressed as means (standard deviation). TG was described by median and inter-quartile range.

Abbreviations: NWNT, normal waist normal triglyceride; HTG, hypertriglyceridemia; EW, enlarged waist circumference; HTGW, hypertriglyceridemic-waist; BMI, body mass index; WC, waist circumference; SBP, systolic blood pressure; DBP, diastolic blood pressure; TC, total cholesterol; LDL-C, low density lipoprotein-cholesterol; HDL-C, high density lipoprotein-cholesterol; TG, triglyceride; FPG, fasting plasma glucose.

^a^ Comparison among four groups.

^b^ NS = no significant, P value >0.05.

^**#**^ Compared with NWNT group, P <0.05.

^**&**^ Compared with HTG group, P <0.05.

^**$**^ Compared with EW group, P <0.05.

Note: NWNT was defined as serum triglyceride concentration <1.7 mmol/L and waist circumference <90 cm for men and <80 cm for women; HTG was defined as serum triglyceride concentration ≥1.7 mmol/L and waist circumference <90 cm for men and <80 cm for women; EW was defined as serum triglyceride concentration <1.7 mmol/L and waist circumference ≥ 90 cm for men and ≥ 80 cm for women; HTGW was defined as serum triglyceride concentration ≥1.7 mmol/L and waist circumference ≥ 90 cm for men and ≥ 80 cm for women.

**Figure 1:**
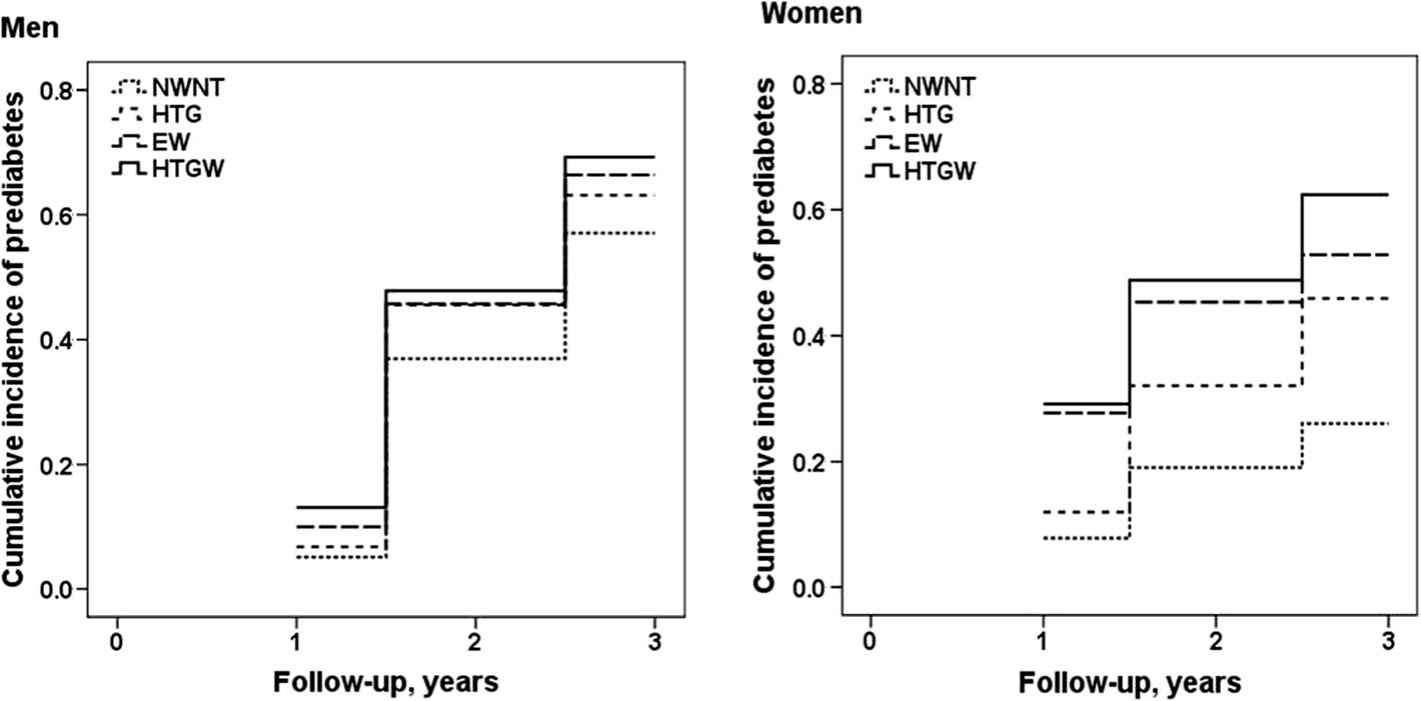
**Cumulative incidence of prediabetes stratified by the 4 phenotype groups for men and women. **Note: NWNT (normal waist normal triglyceride) was defined as serum triglyceride concentration <1.7 mmol/L and waist circumference <90 cm for men and <80 cm for women; HTG (hypertriglyceridemia) was defined as serum triglyceride concentration ≥1.7 mmol/L and waist circumference <90 cm for men and <80 cm for women; EW (enlarged waist) was defined as serum triglyceride concentration <1.7 mmol/L and waist circumference ≥ 90 cm for men and ≥ 80 cm for women, HTGW (hypertriglyceridemic-waist) was defined as serum triglyceride concentration ≥1.7 mmol/L and waist circumference ≥ 90 cm for men and ≥ 80 cm for women.

**Figure 2:**
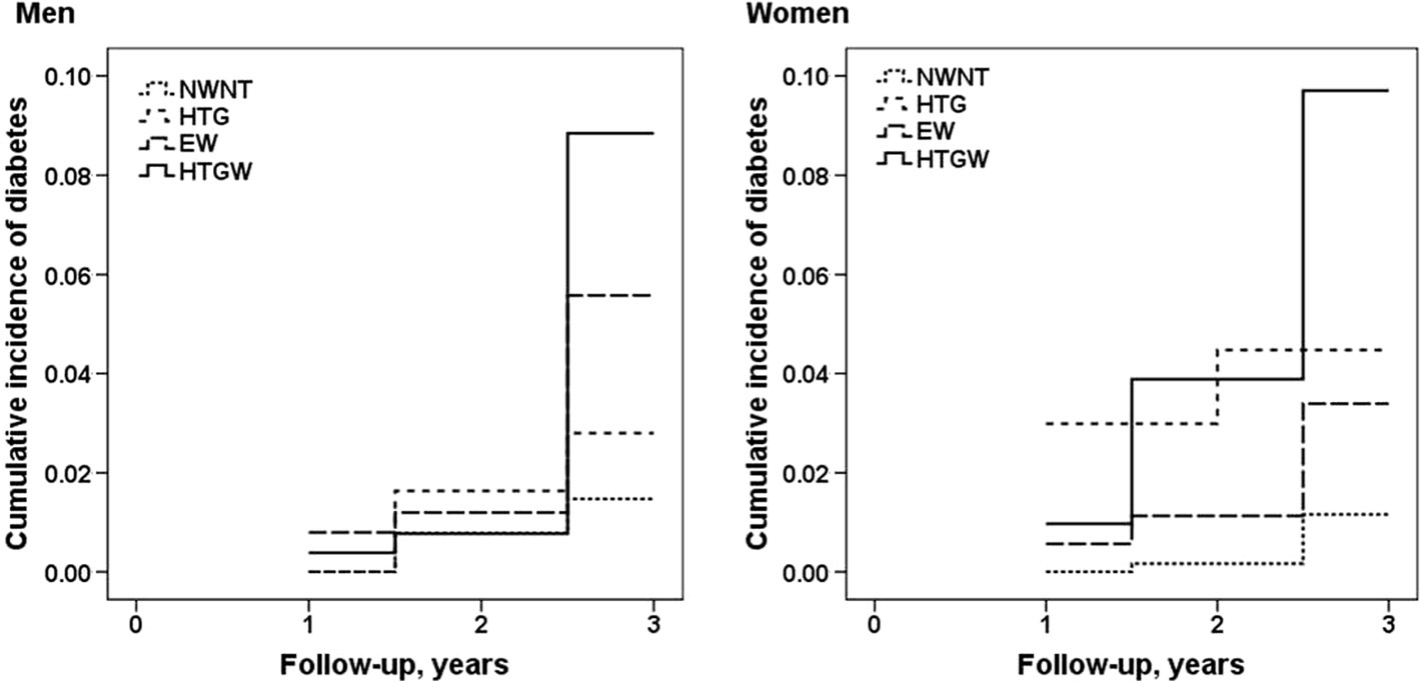
**Cumulative incidence of diabetes stratified by the 4 phenotype groups for men and women. **Note: NWNT (normal waist normal triglyceride) was defined as serum triglyceride concentration <1.7 mmol/L and waist circumference <90 cm for men and <80 cm for women; HTG (hypertriglyceridemia) was defined as serum triglyceride concentration ≥1.7 mmol/L and waist circumference <90 cm for men and <80 cm for women; EW (enlarged waist) was defined as serum triglyceride concentration <1.7 mmol/L and waist circumference ≥ 90 cm for men and ≥ 80 cm for women, HTGW (hypertriglyceridemic-waist) was defined as serum triglyceride concentration ≥1.7 mmol/L and waist circumference ≥ 90 cm for men and ≥ 80 cm for women.

The cumulative incidences of prediabetes and diabetes among the 4 phenotype groups are shown in Figure 1 and Figure 2. Compared with the NWNT group, those in the HTGW group had higher incidences prediabetes and diabetes. Moreover, the cumulative incidences of prediabetes and diabetes in the HTG group and the EW group were higher than in the NWNT group. These findings were found for both men and women. 

The hazard ratios for the risk of prediabetes and diabetes before and after adjustment for age, BMI, SBP, TC, HDL-C and LDL-C were shown in Table 3 and Table 4. Compared with the participants in the NWNT group, those in the HTGW group had an unadjusted hazard ratio (HR) (95% confidence interval [CI]) of 1.34 (1.13-1.58) in men and 2.87 (2.14-3.85) in women for prediction of prediabetes. After adjusting for covariates, the relationship remained significant (HR, 1.51; 95% CI, 1.04-2.19; P= .029) in women only (Table 3). As shown in Table 4, participants in the HTGW group had an unadjusted HR (95% CI) of 6.01 (3.14-11.52) in men and 8.60(3.28-22.60) in women for prediction of diabetes compared to those in the NWNT group. This relationship remained significant after adjustment for covariates. Hypertriglyceridemic-waist phenotype was associated with a 4.5- and 4.6-fold increase in diabetes risk for men and women, respectively. Although the enlarged waist phenotype group had a 3.4-fold increase in diabetes risk only in men after adjustment for covariates, the HR was lower than in the HTGW group (4.46). 

**Table 3:** Risk of prediabetes by the 4 phenotype groups for men and women

**Phenotype group**	**No. of person-years**	**No. of prediabetes**	**Unadjusted**			**Adjusted**^**a**^		
			**HR**	**95% CI**	**P value**	**HR**	**95% CI**	**P value**
Men								
NWNT	2632	578	Reference			Reference		
HTG	1075	268	1.15	0.99-1.33	0.061	1.07	0.92-1.26	0.264
EW	619	165	1.24	1.04-1.47	0.015	0.95	0.78-1.16	0.678
HTGW	408	179	1.34	1.13-1.58	0.000	1.01	0.82-1.24	0.898
Women								
NWNT	1650	157	Reference			Reference		
HTG	172	30	1.83	1.24-2.70	0.003	1.42	0.96-2.12	0.083
EW	404	93	2.41	1.87-3.12	0.000	1.61	1.17-2.21	0.003
HTGW	230	63	2.87	2.14-3.85	0.000	1.51	1.04-2.19	0.029

^a^ Adjusted for age, body mass index, systolic blood pressure, total cholesterol and high density lipoprotein-cholesterol.

Abbreviations: NWNT, normal waist normal triglyceride; HTG, hypertriglyceridemia; EW, enlarged waist circumference; HTGW, hypertriglyceridemic-waist; HR, hazard ratio; 95% CI, 95% confidence interval.

Note: NWNT was defined as serum triglyceride concentration <1.7 mmol/L and waist circumference <90 cm for men and <80 cm for women; HTG was defined as serum triglyceride concentration ≥1.7 mmol/L and waist circumference <90 cm for men and <80 cm for women; EW was defined as serum triglyceride concentration <1.7 mmol/L and waist circumference ≥ 90 cm for men and ≥ 80 cm for women; HTGW was defined as serum triglyceride concentration ≥1.7 mmol/L and waist circumference ≥ 90 cm for men and ≥ 80 cm for women.

**Table 4:** Risk of diabetes by the 4 phenotype groups for men and women

**Phenotype group**	**No. of person-years**	**No. of diabetes**	**Unadjusted**			**Adjusted**^**a**^		
			**HR**	**95% CI**	**P value**	**HR**	**95% CI**	**P value**
Men								
NWNT	3055	15	Reference			Reference		
HTG	1277	12	1.91	0.90-4.01	0.094	1.55	0.69-3.49	0.295
EW	748	14	3.80	1.84-7.88	0.000	3.37	1.43-7.96	0.005
HTGW	777	23	6.01	3.14-11.52	0.000	4.46	1.88-10.60	0.001
Women								
NWNT	1811	7	Reference			Reference		
HTG	197	3	3.96	1.02-15.31	0.046	3.66	0.88-15.31	0.076
EW	528	6	2.95	0.99-8.77	0.052	1.71	0.46-6.44	0.426
HTGW	304	10	8.60	3.28-22.60	0.000	4.64	1.20-17.97	0.017

^a^ Adjusted for age, body mass index, systolic blood pressure, total cholesterol and high density lipoprotein-cholesterol.

Abbreviations: NWNT, normal waist normal triglyceride; HTG, hypertriglyceridemia; EW, enlarged waist circumference; HTGW, hypertriglyceridemic-waist; HR, hazard ratio; 95% CI, 95% confidence interval.

Note: NWNT was defined as serum triglyceride concentration <1.7 mmol/L and waist circumference <90 cm for men and <80 cm for women; HTG was defined as serum triglyceride concentration ≥1.7 mmol/L and waist circumference <90 cm for men and <80 cm for women; EW was defined as serum triglyceride concentration <1.7 mmol/L and waist circumference ≥ 90 cm for men and ≥ 80 cm for women; HTGW was defined as serum triglyceride concentration ≥1.7 mmol/L and waist circumference ≥ 90 cm for men and ≥ 80 cm for women.

In addition, we calculated the hazard ratios for the risk of prediabetes and diabetes according to baseline values of waist circumference and serum triglyceride and reached the same outcomes. Compared with the participants in the NWNT group, those in the HTGW group had an unadjusted HR (95% CI) of 1.51 (1.27-1.81) in men and 2.89 (2.15-3.91) in women for prediction of prediabetes. After adjusting for covariates, the relationship remained significant (HR, 1.54; 95% CI, 1.12-2.109; P= .0007) in women only, not in men (HR, 1.15; 95% CI, 0.93-1.44; P= .196). Similarly, compared with the participants in the NWNT group, those in the HTGW group had an unadjusted HR (95% CI) of 5.80 (3.12-10.78) in men and 8.93 (2.92-27.29) in women for prediction of diabetes. After adjusting for covariates, the relationship remained significant in both men (HR, 3.77; 95% CI, 1.63-8.69; P= .002) and women (HR, 6.08; 95% CI, 1.36-27.12; P= .017).

## Discussion

The Chinese population has a relatively lower prevalence of diabetes and prediabetes compared to that in many industrialized countries, but there is no doubt that the prevalence of diabetes and prediabetes has been increasing rapidly in recent years in China. The concept of the hypertriglyceridemic-waist phenotype proposed by Lemieux and colleagues [10] has suggested that this simple phenotype could be a useful marker of metabolic abnormalities. Previous studies have reported the association among the HTGW phenotype, cardiometabolic risk and Type 2 diabetes mellitus. A recent study in the Chinese population has demonstrated that the phenotype of visceral obesity HTGW was closely associated with prediabetes and diabetes [16]. Also in Canadian Aboriginals where the HTGW was associated with a five-fold increased risk for the development of Type 2 diabetes mellitus [20]. Our results are consistent with the previous studies and provide evidence from a Chinese cohort that the hypertriglyceridemic-waist phenotype is a simple and inexpensive marker to identify patients with intraabdominal obesity who have a deteriorated cardiometabolic risk profile and are thus at increased risk of diabetes.

It is well known that the direct and precise measurement of visceral adipose tissue can only be possible with the use of imaging techniques such as computed tomography and magnetic resonance imaging. It has been reported that the occurrence of hypertriglyceridemic waist phenotype in subjects with type 2 diabetes identifies a subset with greater degree of visceral adiposity including visceral adipose tissue (VAT) and subcutaneous adipose tissue (SAT), even in the presence of type 2 diabetes, an elevated waist circumference, by itself, does not identify subjects with the highest accumulation of visceral fat [9]. Thus, simultaneous measurement of fasting triglycerides and waist circumference (HTGW) is a useful approach to identify subjects with the greatest amount of visceral fat compared with the phenotypes of EW and HTG [21,22]. Indeed several studies had indicated that HTGW as the phenotype of visceral obesity is more closely associated with diabetes rather than EW [23,24]. Interestingly, in the present study, HTGW remained significantly associated with the risk of diabetes after adjustment for covariates. In addition, higher levels of fasting glucose were found in Chinese adults with HTGW compared with the counterparts of EW or HTG.

Hypertriglyceridemic-waist phenotype would first be considered as a surrogate marker for a particularly deleterious dyslipidemic, insulin-resistant and pro-inflammatory profile, associated with abdominal obesity. Excess visceral abdominal fat mass is associated with increased release of free fatty acids into the circulation, which in turn can inhibit glucose uptake and oxidation by muscle and other organs [25]. Increased secretion of insulin may temporarily compensate for these alterations, but the chronic presence of triggering mechanisms may lead to dysfunction of these cells, thereby promoting diabetes [26].

Moreover, it was observed that HTGW increased risk of prediabetes occurrence in both men and women. However, adjustments for common CVD risk factors make this increased risk disappeared in men. The HTGW phenotype is strongly associated with the predicted development of prediabetes in women. Hypertriglyceridemic-waist phenotype could be a marker of abnormal lipid overflow because of a defect of adipose tissue in cleaning up and storing the excess triglycerides due to over nutrition and lack of physical activity [27]. The notion that “lipid over accumulation” has different importance for atherogenesis and CVD in the both genders, which arises from observations that elevated triglyceride is an useful indicator for the risk of poor outcomes in women but less in men [28,29]. Although there was the evidence to show a combination of TG levels and waist circumference as lipid accumulation product predicted CVD risk in men [30], HTGW may not predict CVD risk among men who are already at increased risk for CVD. Similarly, our results also indicated that HTGW phenotype may not predict the risk of prediabetes in men and is only useful for women to predict prediabetes. Further research will be needed as the small sample size and the short follow-up period in the current study.

Our study has several limitations including short follow-up period, small sample size, and lack of information on lifestyle and dietary intake. In addition, oral glucose tolerance tests were not performed at baseline, possibly leading to an underestimate of the incidence of prediabetes and diabetes and the impact of HTGW on the risk of these conditions. However, it is important to observe a strong association between HTGW and the risk of developing diabetes though lacking in the oral glucose tolerance test.

## Conclusions

In conclusion, the hypertriglyceridemic-waist phenotype can be used as a simple screening approach to predict diabetes. By using this approach, it is possible to identify individuals at high-risk for diabetes, which is of great significance in reducing the incidence of diabetes among Chinese urban adults.

## Competing interests

The authors declare that they have no competing interests.

## Authors’ contributions

MZ performed study design and statistical analyses, interpreted results and drafted the manuscript. GH contributed to designing the study and interpreting results and helped to draft the manuscript. YG and XW participated in the study design, statistical analysis and interpretation of data. HC participated in and carried out the field work. DL and ZZ participated in interpreting results and technical support. All authors read and approved the final manuscript.

## Pre-publication history

The pre-publication history for this paper can be accessed here:

http://www.biomedcentral.com/1471-2458/12/1081/prepub
